# Tackling the translational challenges of multi-omics research in the realm of European personalised medicine: A workshop report

**DOI:** 10.3389/fmolb.2022.974799

**Published:** 2022-10-13

**Authors:** Emanuela Oldoni, Gary Saunders, Florence Bietrix, Maria Laura Garcia Bermejo, Anna Niehues, Peter A. C. ’t Hoen, Jessica Nordlund, Marian Hajduch, Andreas Scherer, Katja Kivinen, Esa Pitkänen, Tomi Pekka Mäkela, Ivo Gut, Serena Scollen, Łukasz Kozera, Manel Esteller, Leming Shi, Anton Ussi, Antonio L. Andreu, Alain J. van Gool

**Affiliations:** ^1^ European Infrastructure for Translational Medicine (EATRIS), Amsterdam, Netherlands; ^2^ Biomarkers and Therapeutic Targets Group, Ramon and Cajal Health Research Institute (IRYCIS), Madrid, Spain; ^3^ Translational Metabolomic Laboratory, Department of Laboratory Medicine, Radboud Institute for Molecular Life Sciences, Radboud University Medical Center, Nijmegen, Netherlands; ^4^ Center for Molecular and Biomolecular Informatics, Radboud Institute for Molecular Life Sciences, Radboud University Medical Center, Nijmegen, Netherlands; ^5^ Department of Medical Sciences, Molecular Precision Medicine and Science for Life Laboratory, Uppsala University, Uppsala, Sweden; ^6^ Institute of Molecular and Translational Medicine, Faculty of Medicine and Dentistry, Palacky University and University Hospital in Olomouc, Olomouc, Czechia; ^7^ Institute for Molecular Medicine Finland FIMM, University of Helsinki, Helsinki, Finland; ^8^ iCAN Digital Precision Cancer Medicine Flagship, University of Helsinki, Helsinki, Finland; ^9^ HiLIFE-Helsinki Institute of Life Science, University of Helsinki, Helsinki, Finland; ^10^ CNAG-CRG, Centre for Genomic Regulation (CRG), Barcelona Institute of Science and Technology (BIST), Barcelona, Spain; ^11^ ELIXIR Hub, Hinxton, United Kingdom; ^12^ Biobanking and BioMolecular Resources Research Infrastructure-European Research Infrastructure Consortium (BBMRI-ERIC), Graz, Austria; ^13^ Josep Carreras Leukemia Research Institute (IJC), Badalona, Spain; ^14^ Centro de Investigacion Biomedica en Red Cancer (CIBERONC), Madrid, Spain; ^15^ Institucio Catalana de Recerca i Estudis Avançats (ICREA), Barcelona, Spain; ^16^ Physiological Sciences Department, School of Medicine and Health Sciences, University of Barcelona (UB), Barcelona, Spain; ^17^ State Key Laboratory of Genetic Engineering, School of Life Sciences and Human Phenome Institute, Fudan University, Shanghai, China

**Keywords:** personalised medicine, translational medicine, multi-omics, EU initiatives, research infrastructures, bottlenecks in health data

## Abstract

Personalised medicine (PM) presents a great opportunity to improve the future of individualised healthcare. Recent advances in -omics technologies have led to unprecedented efforts characterising the biology and molecular mechanisms that underlie the development and progression of a wide array of complex human diseases, supporting further development of PM. This article reflects the outcome of the 2021 EATRIS-Plus Multi-omics Stakeholder Group workshop organised to 1) outline a global overview of common promises and challenges that key European stakeholders are facing in the field of multi-omics research, 2) assess the potential of new technologies, such as artificial intelligence (AI), and 3) establish an initial dialogue between key initiatives in this space. Our focus is on the alignment of agendas of European initiatives in multi-omics research and the centrality of patients in designing solutions that have the potential to advance PM in long-term healthcare strategies.

## Introduction

### Definition of personalised medicine and -omics technologies

The ‘Personalised Medicine’ (PM) field has evolved rapidly over recent years, and now plays an increasingly important role in disease prevention, diagnosis, prognosis, and the unearthing of novel therapeutics. The European Commission recently defined PM as: “a *medical model using characterization of individuals’ phenotypes and genotypes (e.g., molecular profiling, medical imaging, lifestyle data) for tailoring the right therapeutic strategy for the right person at the right time, and/or to determine the predisposition to disease and/or to deliver timely and targeted prevention”* (European Commission, 2021)*.* This definition has also been adopted by the European PERMIT project (Banzi et al., 2020).

The application of PM has the clear potential to aid routine clinical decision making processes based on (an) individual patient profile(s) and condition(s) in order to minimise harmful side effects, ensure a more successful outcome, assure more efficient patient management, and at the same time provide an economic advantage (Goetz and Schork, 2018; Kisor and Ehret, 2020). A strong contribution to this comes from new molecular biomarker analysis technologies, often summarised under the term -“omics” (e.g., genomics, transcriptomics, proteomics, metabolomics, radiomics, and lipidomics) (Karczewski and Snyder, 2018; Krassowski et al., 2020). Following landmark observations of genetic variants for use as stratification biomarkers to select best responding patients, including Her-2 amplification in breast cancer Pegram et al. (1998) and BRAF mutation in melanoma Davies et al. (2002), multiple examples of omics impact in PM were described, such as in oncology van 't Veer et al. (2002), diabetes Chen et al. (2012), inflammatory bowl disease Lloyd-Price et al. (2019) and rare neurometabolic disease Tarailo-Graovac et al. (2016). The resulting data enable scientists and healthcare professionals to obtain mechanistic insights in a patients’ disease state and determine the correct course of action (Chen and Snyder, 2013).

However, the integration of multiple different types of -omics data to identify composite biomarker signatures is a current bottleneck in PM. This integration is further complicated when we consider the integration with other data such as imaging data, phenotypic data, medical data (Electronic Health Records (EHRs) and patient-related outcomes (Abul-Husn and Kenny, 2019). This level of integrated multi-modal omics and phenotypic (and/or health) data application facilitates a more precise understanding of disease biology. When disease mechanisms are better understood, drug/therapeutic target identification and the selection of course of treatment for a specific subgroup of patients is possible (Karczewski and Snyder, 2018; Krassowski et al., 2020). The integration of multi-omics and multi-modal data marks a significant step closer to PM (Wenk, 2005; Aslam et al., 2017; Li et al., 2017; Kalisky et al., 2018; Olivier et al., 2019), although many challenges remain before the further development and implementation of these integrated data in routine clinical care.

## European workshop to discuss PM potentials, bottlenecks and challenges, and propose solutions

The EATRIS-Plus project, funded by the EU’s research and innovation funding programme Horizon 2020, aims to build capabilities and deliver innovative scientific tools to support the long-term sustainability of EATRIS as one of Europe’s key PM research infrastructures.

As part of this project, the EATRIS-Plus Multi-omics Stakeholder Group was established in 2020 with the intention of facilitating best practices for omics research and bringing better alignment in goals and objectives in large-scale multi-omics initiatives across Europe and beyond. Moreover, the group seeks to become a key opinion leader group in PM.

The EATRIS-Plus Multi-omics Stakeholder Group held its first virtual meeting on 4 March 2021. The group brings together close to 20 experts from world-leading European institutions and associated with EATRIS-Plus. Further expert members include key stakeholders in the multi-omics landscape, such as Ivo Gut representing the EASI Genomics initiative, Manel Esteller from the International Human Epigenome Consortium, Katja Kivinen from the 1 + MG initiative, Esa Pitkänen and Tomi Mäkelä from the iCAN Digital Precision Cancer Medicine project, and Leming Shi (Fudan University, China) representing the International Human Phenome Consortium. In addition to EATRIS, a further two European Research Infrastructures were represented by Lukasz Kozera and Michaela Mayrhofer from BBMRI and Jennifer Harrow and Serena Scollen from ELIXIR.

The focus of the workshop was to explore common bottlenecks and start a dialogue around potential areas of collaboration across participating organisations. As mentioned above, the overall aim of this multidisciplinary and cross-institutional working group is to become a European reference group for fully implementing PM across Europe.

This first publication from the group reports the main conclusions on the identification of current bottlenecks, pitfalls and potential solutions in multi-omics research in support of PM.

## Bottlenecks in the application of multi-omics to PM

### Moving beyond genomics to integrated multi-omics and multi-modal complex biomarker generation

Diseases are caused by a complex combination of genetic and environmental factors. As a consequence, the uncovering of precise molecular processes by which these factors result in the disease phenotype(s) is vital, yet difficult.

Historically, genomics (e.g., using an individual patient’s genotypic information) laid the foundation for PM (Sadee, 2011). PM has been successfully applied in the areas where strong genetic drivers provide an excellent platform for developing personalised approaches, for example in oncology (Berger and Mardis, 2018) and rare diseases (Alves et al., 2018). Indeed, there are nowadays many reported successes in the application of genomics to clinical care, and this portfolio of success continues to grow (Shendure et al., 2019).

However, the application of PM for other areas of disease where solely genetic factors are less of a driver, such as neurological or metabolic disorders, can be considered as in its infancy. Analysis and application of PM that goes beyond genomics alone and passes through the development and validation of integrated multi-omics biomarker signatures including all the biological layers (effectors and regulators) rather than small sets of putative biomarkers has been demonstrated (Prasser et al., 2018; Glaab et al., 2021). A significant bottleneck, a significant bottleneck in the application to PM remains the required multi-modal data integration that can exploit and integrate multiple molecular and clinical data types in order to improve our understanding of disease mechanisms, stratify patients and inform clinicians about optimised strategies for therapeutic intervention.

### New technologies, new challenges, and digital health

A key element discussed during the first EATRIS-Plus Multi-omics Stakeholder Group was the role of artificial intelligence (AI) in boosting the use of multi-omics in the PM domain. AI can be harnessed to deconvolute high-dimensional data from multi-omics data profiling and resolve molecular profiles that are indicative of treatment response and/or potential drug toxicity. However, examples of the application of AI in omics analyses are scarce which optimally need sufficient multi-scale, multi-modal and longitudinal omics data to reasonably capture relationships that may exist between input and output features. Also, significant challenges are faced in the application of AI to the PM domain in the areas of interoperability, data quality and result reproducibility.

Similar challenges are faced by digital health, being a promising, multidisciplinary approach under development encompassing the use of medical technologies (wearable devices, digital healthcare programs, etc), that permit disease monitoring, management, health risk assessment and/or prevention (Jandoo, 2020). Digital health offers great potential in PM. It improves medical outcomes and enhances efficiency, empowering patients to make better-informed decisions about their own health and providing new options for the prevention, early diagnosis, and management of chronic conditions outside of traditional health care settings (Jandoo, 2020). Additionally, digital health is a potential source of data that can be useful for designing public health policies and epidemiological programs with impact in PM.

### Data standardisation to enable multi-modal integration and AI supported drug modelling

Data modelling can predict many patient characteristics, but its prediction accuracy depends on the quantity and quality of available data, as well as the interoperability of the tools (algorithms, code) used. Using AI to build effective evidence-based decisions requires the collection of significant volume of complex standardised data (multi-omics, imaging, EHRs, etc.) that need to be reliable. The volume of biomedical data being collected has increased exponentially over the past years, but these data are not always readily available for AI-based approaches. This is partly due to the sensitive nature of clinical phenotype data and to the difficulties in obtaining standardised and structured experimental datasets and information from EHRs and research databases (Panahiazar et al., 2015; Abul-Husn and Kenny, 2019; Conesa and Beck, 2019).

Mathematical models should be both flexible and dynamic; as data is continuously provided the model should improve and be more accurate. However, data that is not of high quality will produce results that are not actionable or insightful, and that can even be misleading and useless in clinical practice. Therefore, high-quality multi-omics, clinical, and epidemiological data are fundamental for generating, establishing, and sustaining algorithms that are sufficient for application.

In order to increase quality, standardisation, and reusability of scientific data, and specifically for these data to be machine actionable, the FAIR principles were published in 2016 (Wilkinson et al., 2016). For data to be truly FAIR (Findable, Accessible, Interoperable, Reusable), the principles need to be applied to data at source, including information relating to samples, experimental methods, and data analyses. This is imperative to ensure the required results’ reproducibility needed for the application of AI in the field of PM and to promote the reuse of multi-omics data in patient management (Wilkinson et al., 2016; Abul-Husn and Kenny, 2019; FAIR, 2021).

The FAIR data principles have provided a valuable route forward to the standardisation of data enabling the application of AI in PM. However, due to varying data qualities and multiple different standards used across the landscape, heterogeneity and reduced interoperability, e.g., a timely and secure access, is still common. Integration and use of EHR data so that it can be used to optimise health outcomes for individuals and populations is difficult, and the true application of multi-modal data to PM remains a challenge. Wider adoption of health data standards and models such as the Fast Healthcare Interoperability Resources (FHIR), the Clinical Data Interchange Standards Consortium (CDISC) and the Observational Medical Outcomes Partnership (OMOP), and continuing efforts to map these data models to each other, is needed (Fischer et al., 2020).

### Variability in omics data at source

A key factor for the quality and reusability of multi-omics and clinical data is the availability and feasibility of relevant quality assurance (QA) and quality control (QC) schemes for laboratories in the field. Limited participation in QA/QC schemes due to budget or other constraints leads to decreased harmonisation of sample and data processing methods across laboratories, potentially resulting in lower reliability of analytical results (Freedman et al., 2015). Although standardisation of genomics data is improving (Endrullat et al., 2016; Lubin et al., 2017; Corpas et al., 2018) for other -omics data and EHRs there is a distinct lack of common standards and/or reference benchmark values for assessing complex tests and for determining clinical validity and utility of, for example, biomarkers (Quackenbush, 2004; Simons, 2018; Veenstra, 2021).

Many multi-omics focussed initiatives, such as the EATRIS-Plus project are aimed at tackling the challenges of data interoperability and increasing data FAIRness with the delivery of standard operating procedures (SOPs) that are ready to be implemented by the scientific and clinical communities, enabling standardisation among methods and technical controls in order to increase results reproducibility and improve reliability of the techniques.

An additional critical consideration for the application of multi-omics and clinical data to PM is population diversity. As the biological determinants of health are strongly influenced by environmental and sociocultural factors, and European populations are characterised by genetic and biological diversity, population-tailored reference values for multi-omic and clinical data are required. Currently there is a need of large cohorts representing human population genotypes diversity that can be analysed in order to deliver accurate reference values. The EATRIS-Plus project is aiding this situation by providing a proof of concept with a focus on the Caucasian population (EATRIS, 2022a).

### Data privacy and regulatory aspects, and economic implications

Many data stewardship aspects provide significant challenges to data privacy, for example in the areas of data harmonisation and data curation, use of Common Data Models, standardised nomenclature and data transfer specifications. Such challenges and data management decisions have critical implications for data access both within and outside of jurisdictional regions and, for example, on design, creation and implementation of extract transform load workflows enabling data source maintenance and governance, quality assessment and testing.

Additionally, general concerns around data privacy and regulatory compliance-related restrictions as well as ethical and legal aspects must not be overlooked. When working with multi-omics and/or clinical data there are multiple data security, ethical, and personal information barriers that can present potential roadblocks (Knowles et al., 2017; Adamo et al., 2018; Adamo et al., 2020). Moreover, each European country has its own national implementations of General Data Protection Regulation (GDPR) for processing personal data (Vlahou et al., 2021).

The regulatory framework of PM is still emerging, and the lack of clear regulation continues to discourage investment in the field, especially since developing and implementing personalised approaches is costly - as described in the 2020 report on the current state of PM from the Personalised Medicine Coalition (PMC). In fact, coverage and payment policies both in the public and private sectors play an important role in ensuring patient access and encouraging continued innovation. To tackle the rising health care costs, often policy makers and payers do not promote PM (Kisor and Ehret, 2020). However, the costs of PM could be justified as an investment: PM approaches supporting disease prevention and identifying best therapeutic treatment will likely improve patient life quality and reduce cost in the healthcare management (Gavan et al., 2018). In fact, PM could be considered as social responsibility since most of us will become patients in our lifetimes.

### Tackling the challenges for implementation of PM in routine clinical care

As for all novel technologies, for assuring an effective implementation in clinical care a series of factors need to be taken in account: 1) the benefits, 2) the risks, 3) associated ethical and social aspects and 4) room for innovation. The integration of these four components requires the strong and effective communication between all stakeholders involved in the PM pipeline in order to successfully tackle all challenges. In particular, patients should be placed at the centre and empowered working in concert with researchers, clinicians, industry, and regulators. Patients should be directly involved in research, actively participating in projects, and they should take control as much as possible of their treatment.

It is true to say that healthcare professionals cannot manage a patient properly without taking into account his or her value or the patient’s lifestyle, an important aspect of PM. This is not always straightforward and requires a cultural change that has already started. EATRIS is contributing to this transformation via different initiatives involving the European Patients’ Academy on Therapeutic Innovation (EUPATI) and the European Patient Forum (EPF) (EATRIS, 2021; EATRIS, 2022b; EATRIS, 2022c).

Furthermore, the EATRIS Data Pillar, a key structure of the EATRIS strategy, is devoted not only to the harmonisation of data management and application but also to the unveiling and/or designing of new pathways for uncovering novel therapeutic strategies, with patients at the centre of considerations, continuing to ensure patient privacy. This is reflected in the project portfolio of EATRIS data, empowering the community to drive towards data standardisation, validation, and reproducibility to deliver transformative revolution in the translational medicine domain (Table 1).

**TABLE 1:** EATRIS project portfolio in the Data field.

Project	Scope	Goals
Beyond 1 million Genomes (B1MG)	Access to the genome information of at least one million European citizens for joint European research by 2022	• To make the genome information of at least one million European citizens accessible for joint European research as if it were one large cohort, while the data will be made accessible using a federated infrastructure
EATRIS-Plus	To build further capabilities and deliver innovative scientific tools to support the long-term sustainability strategy of EATRIS as one of Europe’s key research infrastructures for PM	• To develop a multi-omic toolbox to support cross omic analysis and data integration in clinical samples
HealthyCloud	To support the creation of a European Health Data Space	• To deliver a Strategic Agenda including a Ready-to-implement Roadmap for the European Health Data Space ecosystem
		• The project has been organized around four fundamental objectives that cover:
		• interactions with stakeholders to ensure their voices are included as part of the Strategic Agenda
		• the inclusion of Ethical, Legal and Societal aspects in the design of the future Health Research and Innovation Cloud (HRIC) ecosystem
		• the sustainable access, use and re-use of health-related data considering a progressive adoption of the FAIR principles
		• the technological solutions in terms of computational facilities and mechanisms to enable distributed health data analysis across Europe
EOSC-Life	To create an open collaborative digital space for life science	• To publish ‘FAIR’ data and a catalogue of services provided by participating RIs for the management, storage and reuse of data in the European Open Science Cloud (EOSC)
		• To implement workflows across disciplines and address the needs of interdisciplinary science
		• To address the data policies needed for human research data under GDPR
EOSC-Future	To demonstrate an operational EOSC Platform (‘System of Systems’) with an integrated execution environment consisting of data, professionally provided services, and open research products and infrastructure that will be accessed and used by the European researchers	• To realise a EOSC-Core and EOSC-Exchange with interoperable data and resources
		• To allow the integration of data and resources from the Science Cluster communities into the EOSC Platform
		• To involve users in the co-design and implementation of the EOSC Platform
BY-COVID	To connect well-established data resources and deliver access to heterogeneous yet interlinked and organised data across domains and jurisdictions via the components of the COVID-19 Data Platform (https://www.covid19dataportal.org/)	• To create a flexible and interlinked core of FAIR data capable of addressing the constantly evolving questions during a pandemic

Moreover, the adaption of the regulatory, ethical and legal landscape for facilitating the exploitation of patient data in the context of PM is simultaneously occurring across many large European organisations. For example, the EATRIS regulatory service and support centre is available to guide key stakeholders through this complex world, especially for complex and hybrid products for which clear regulatory guidance may not be available.

## European cooperation for tackling the challenges of multi-omics in the realm of PM

In order to overcome the aforementioned challenges of PM implementation several national and international initiatives are working towards providing solutions in the further development and implementation of multi-omics research (Table 2). However, a strong synergy between such initiatives is needed to further ensure successful outcomes and to tackle any potential defragmentation (Van Gool et al., 2017). Despite this commonly understood need for alignment and cooperation among ongoing initiatives, misalignment of agendas, priorities and deliverables are potential obstacles that must be understood in order to overcome.

**TABLE 2:** European initiatives focused on omics research for PM.

Initiative	Scope	Goals	
EATRIS-Plus	To build further capabilities and deliver innovative scientific tools to support the long-term sustainability strategy of EATRIS as one of Europe’s key research infrastructures for PM	• To develop a multi-omic toolbox to support cross omic analysis and data integration in clinical samples	
		• To drive patient empowerment through active involvement in the infrastructure’s operations	
		• To expand strategic partnerships with research infrastructures and other relevant stakeholders	
1 + Million Genomes (1 + MG)	Access to the genome information of at least one million European citizens for joint European research by 2022	• To make the genome information of at least one million European citizens accessible for joint European research as if it were one large cohort, while the data will be made accessible using a federated infrastructure	
Beyond 1 million Genomes (B1MG)	To make it easier to share human health data around Europe. The project provides coordination and support to 1 + MG.	• To create the infrastructure, the legal guidance and the best practices to enable cross border genetic and phenotypic data access	
International Human Epigenome Consortium (IHEC)	To provide free access to high-resolution References human epigenome maps for normal and disease cell types to the research community	• To coordinate the production of References maps of human epigenomes for key cellular states relevant to health and diseases	
		• To coordinate rapid distribution of the data to the entire research community with minimal restrictions, to accelerate translation of this new knowledge into health and diseases	
		• To coordinate the development of common bioinformatics standards, data models and analytical tools to organize, integrate and display whole epigenomic data generated from this important international effort	
iCAN Digital Precision Cancer Medicine project	To improve outcomes and quality of life of cancer patients	• To integrate tumor molecular profiling and patients’ health data	
		• To improve cancer diagnostics and treatments	
		• To accelerate world-class scientific innovation with the patient in focus	
X-omics	To establish an integrated multi-omics research infrastructure across Netherlands with expertise in molecular biology research (genomics, proteomics, metabolomics, data integration and analysis and their combination)	• To advance X-omics technologies far beyond state-of-art	
		• To realize an integrated X-omics infrastructure in Netherlands	
PERMIT	To develop recommendations for robust and reproducible personalised medicine research	To develop recommendation for • the application and types (supervised or unsupervised) of different stratification algorithms, and the robustness and validation of the stratification methods	
		• translational research establishing a link between data-driven stratification and the choice of treatment options	
		• randomised clinical trials needed to test treatment strategies for each of the identified patient clusters, and to test the added value of the personalised approach vs non-personalised standard of care	
Deutsche COVID-19 OMICS Initiative (DeCOI)	To use NGS-based omics data in COVID-19 research	• To establish an infrastructure addressing short-term, but also mid- and long-term challenges of the current pandemics	
		• To prepare the NGS sector in Germany for future threats	
NeurOmics	To revolutionise diagnostics and develop new treatments for ten major neuromuscular and neurodegenerative diseases	To use the most sophisticated -omics technologies in order to	
		• increase the number of patients with a genetic diagnosis	
		• develop biomarkers for clinical application	
		• improve understanding of pathophysiology and identify drug targets	
		• identify disease modifiers	
		• develop targeted therapies	
		• translate findings to other, related disease groups	
Personal Genome Project United Kingdom	To provide open genome, trait, and health data	• Open access data to enable the timely development of tools for personalised medicine and provide a resource for advancing research	
ICPerMed	To provide a platform to initiate and support communication and exchange on personalised medicine research, funding and implementation	• To contribute to the reasonable and fair implementation of personalised medicine approaches into the health systems for the benefit of patients, citizens and society as a whole	
		• To provide a flexible framework for cooperation between member organisations	
The Personal Health Train Network	To learn from each other’s experiences and solutions. Netherlands PHT network is actively engaged in promoting the FAIR movement, such as the GO FAIR implementation network and committed to the principles for development of the PHT as outlined in the PHT Manifesto	• Promoting data FAIRification	
EASI Genomics	To provide easy and seamless access to cutting-edge DNA sequencing technologies within a framework compliant with ethical and legal requirements, as well as FAIR and secure data management	• To build an infrastructure for enabling omics analyses (genomics, transcriptomics, epigenomics, metagenomics, immunogenomics, etc.)	
IMPACT	Strategic Action designed to offer services to the Spanish R&D&I landscape, oriented to Precision Medicine, through 3 programs	• To promote generation and transfer of high-quality knowledge to the National Health System	
	• Predictive Medicine		
	• Data Science	• To ensure excellence in science and technology	
	• Genomic Medicine	• To assure equity and efficiency in the use of available resources	

In Europe, Research Infrastructures (RIs) can and do facilitate this process. In particular, the Alliance of Medical Research Infrastructures (AMRI, https://eu-amri.org/), consisting of EATRIS (the RI for translational medicine, https://eatris.eu/), ECRIN (the RI for clinical research, https://www.ecrin.org/) and BBMRI (the RI for biobanking https://www.bbmri-eric.eu/) works to support the development of PM by expanding strategic partnerships with relevant stakeholders and expediting interactions. Because of the high-level of participation from countries all over Europe, AMRI allows an alignment of research activities at pan-European scale, and even beyond.

One focus of AMRI is to ensure and implement a common quality framework across the multi-omics domain. The AMRI RIs are already committed to quality in science (Freedman et al., 2015) and lead various actions addressing reproducibility, best practice guidelines, benchmarking, standards and reference materials for generation, sharing and management of multi-omics data and metadata. To date, a lot of effort has been put into enabling genomics as part of the PM pipeline (Sadee, 2011; Berger and Mardis, 2018). In particular, the European Commission driven 1 + Million Genomes initiative (1 + MG) aims to enable access to the genomic information of at least one million European citizens for joint European research as if it were one large cohort, while the data remains safely stored locally (Saunders et al., 2019). To support this effort, the Beyond 1 Million Genomes (B1MG) project is creating the federated infrastructure, including shared legal guidance and best practices for cross-borders data access. Additionally, EASI Genomics, provides easy and seamless access to cutting-edge DNA sequencing technologies to researchers from academia and industry.

**FIGURE 1:**
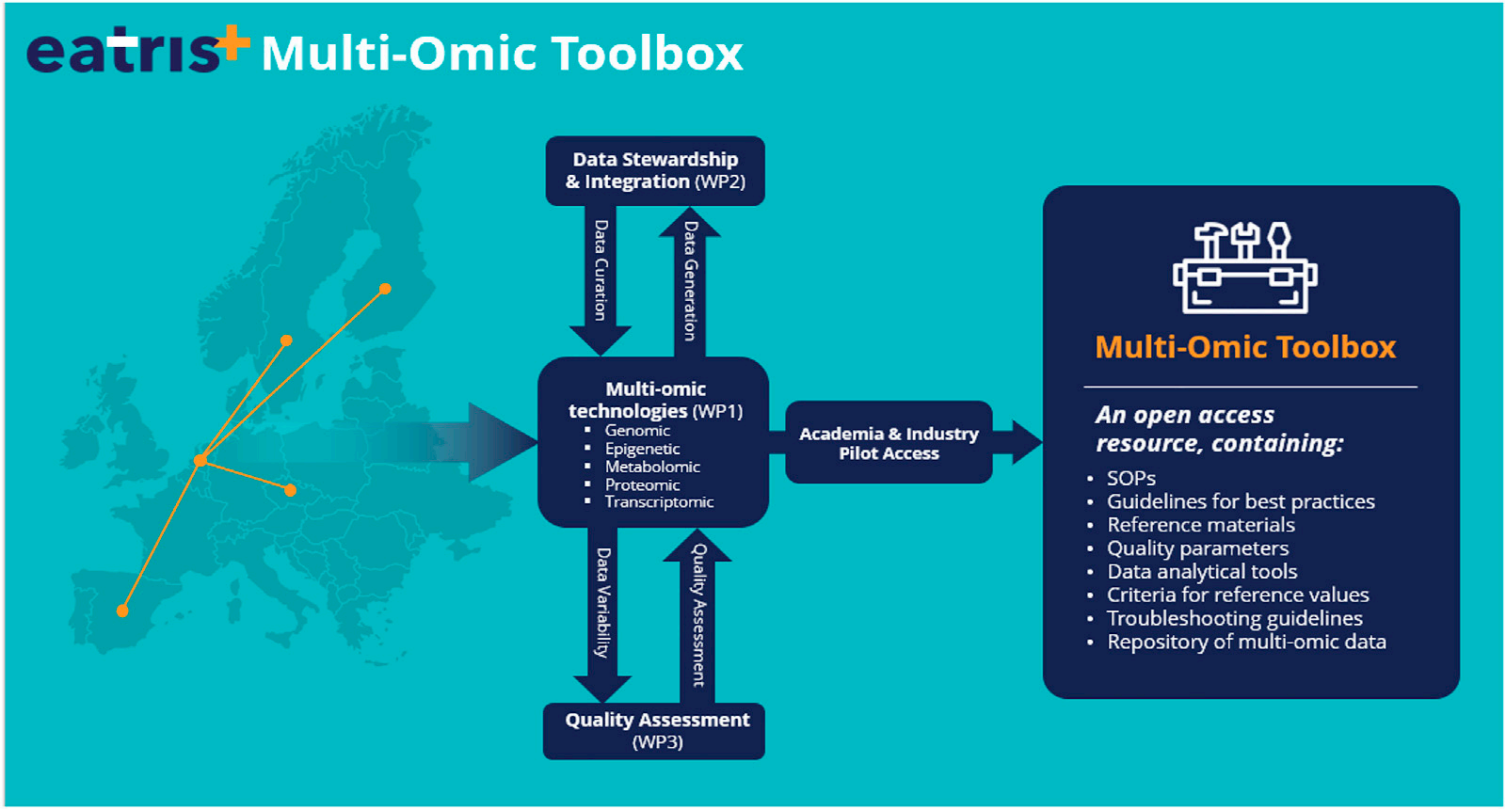
The EATRIS+ multi-omics toolbox. The multi-omics toolbox will be developed and tested with a real-setting demonstrator, an already established cohort of 1,000 healthy individuals in Czechia upon whom genomic sequencing has been already performed. Information available on this healthy individual cohort will be augmented during the project with transcriptomic, proteomic and metabolomic data. By providing such toolbox to the research community, EATRIS-Plus will be the engine to enable high-quality research in the context of patient stratification and accelerate the implementation of Personalised Medicine solutions. EATRIS is the European Infrastructure for Translational Medicine providing services for accelerating biomedical innovation.

However, to go beyond genomics and truly integrate multi-omics in the PM pipeline requires the facilitation of the process of data sharing, federated data analysis and integration for other omics technologies. In this regard, EATRIS-Plus (EATRIS, 2022a) is developing a multi-omic toolbox (Figure 1) to support data integration and joint analysis in clinical samples. By providing such a toolbox to the research community, EATRIS-Plus will act as an engine to enable high-quality research in the context of patient stratification and accelerate the implementation of PM solutions.

There are several similarly focussed initiatives looking to blossom the integration of multi-omics in the PM pipeline at the national level. For example, in Netherlands, the X-omics initiative has established a national research infrastructure consisting of several facilities (genomics, proteomics, metabolomics, and data analysis, integration and stewardship), with the aim of generating data that is FAIR at source and ready for multi-omics integration in a customizable cloud-based digital research environment.

In Finland, the iCAN project is developing a platform for enabling the integration of cutting-edge molecular profiling information from tumours with rich longitudinal health data.

In Spain, the IMPACT initiative in PM are favouring the building of national platforms focused on the implementation of omics techniques and data exploitation in daily clinical practice, through the Health Research Institutes which are members of EATRIS.

Although the outcomes of such initiatives are undoubtably useful and have an impact in PM development and implementation, rapid development also requires fast and flexible ethical, legal and regulatory policy making as well as tackling some technical challenges (Adamo et al., 2018; Misra et al., 2019). To support healthcare providers and patients with new tools, it is crucial to facilitate data access, pilot studies for PM, and incorporate learned lessons into future policymaking.

Common strategies for the implementation of omics technologies in the PM field should be developed in the early stages of projects, even considered during the design of the call topics and proposals, with all relevant stakeholders (researchers, clinicians, patients, regulators, funders), efficiently communicating in order to align agendas and priorities. Stepping into already running processes limits the potential of common understanding and therefore only with a close collaboration from the start, consortia will be able to truly efficiently and effectively support PM development and deliver impactful and transformational solutions. Common objectives, milestones and achievements need to be defined and should always be considered and oriented from the citizens and patient perspectives. Finally, it is fundamental that policymakers engage to bridge the gap between science, medicine and the policy agenda, since we all are eventual patients of our combined European healthcare systems.

## Conclusion

The recent advances in -omics technologies and their integration holds great promise for further development and implementation in the PM pipeline in order to revolutionise European healthcare. This journey is still in its infancy and many complex challenges and issues must be understood and addressed before the true benefits of PM can be seen in full implementation at the clinical setting. The sharing of knowledge on multi-omics capabilities, challenges and potential solutions is imperative for this field to mature and evolve. Here we describe how the EATRIS-Plus Multi-omics Stakeholder Group workshop has brought together relevant stakeholders to work towards PM implementation and commitment to achieve this goal. Key observations are summarized in Table 3.

**TABLE 3:** Summary of challenges and recommendations from the EATRIS + multi-omics workshop (March 2021).

Challenge	Recommendation
Moving beyond genomics	• Communicate and educate on the pros and cons of other omics technologies such as proteomics, metabolomics and lipidomics
	• Develop multi-modal data integration models that showcase the added value of multi-omics approaches in Personlized Medicine
New technologies, new challenges	• Share lessons-learned, failures and successes when evaluating new technologies in Personalized Medicine
	• Evaluate the added value of Artificial Intelligence and Digital health in Personalized Medicine, particularly in combination with multi-omics data
Data standardisation	• Adopt international standards of health data and models including the FAIR principles of data stewardship (e.g., OMOP, FHIR, CDISC)
	• Define criteria for quantity, quality and FAIR levels of data prior to multi-modal data analyses for a specific objective in Personalized Medicine
	• Work with flexible and dynamic mathematical models to adapt to changing data collections in Personalized Medicine
Variability in omics data at source	• Use internationally recognised laboratory standards and standard operating procedures for omics analyses
	• Adopt and apply quality assurance and control schemes for laboratories, such as the EATRIS Certificate of Commitment to Quality
	• Include confounding factors such as population diversity in biological systems in the multi-modal data analysis
Data privacy and regulatory aspects	• Consider ethical, legal, societal aspects when designing multi-omics Personalized Medicine studies
	• Comply with international standards on data security, including the General Data Protection Regulation in personal data
	• Report of the successes and failures of implementations from the European landscape
Implementation of Personalized Medicine in routine clinical care	• Consider well prior to multi-omics Personalized Medicine implementation: 1) the benefits, 2) the risks, 3) associated ethical and social aspects, 4) room for innovation

An improved environment for innovation and for the integration of -omics requires a cultural and educational shift to be embraced by the entire scientific community. One of the biggest challenges will be to convince citizens, patients, healthcare communities and national regulators to allow the sharing of personal, clinical and multi-omics data to enable and accelerate PM.

Data quality and result reproducibility, a good cooperation and communication between multi-omics consortia, and an alignment with the policy agenda, are all essential aspects for facilitating the translation of multi-omics-related discoveries from bench to clinic and only following this approach we will be able to make PM an accessible reality, where the European citizen and patient is at the centre.

## Data Availability

The original contributions presented in the study are included in the article/supplementary material, further inquiries can be directed to the corresponding authors.
